# A Case of Electroencephalography and Machine Learning in Early Diagnosis of Psychotic and Affective Disorders

**DOI:** 10.1192/j.eurpsy.2024.61

**Published:** 2024-08-27

**Authors:** E. Sarisik, D. Popovic

**Affiliations:** ^1^Max Planck Instittute of Psychiatry; ^2^Department of Psychiatry and Psychotherapy, LMU University Hospital, Munich, Germany

## Abstract

**Abstract:**

Electroencephalography (EEG) serves as a non-invasive, cost-effective, and robust tool, directly measuring in-vivo neuronal mass activity with high temporal resolution. Using state-of-the-art machine learning techniques, EEG recordings have the potential to generate in silico biomarkers for severe mental disorders. In this study, we developed EEG-based classification models for schizophrenia and depression taking into account physiological and pathological aging processes.

From a cohort (N=735, 51.6% male) that is acquired in LMU Hospital, Department of Psychiatry and Psychotherapy, comprising healthy control individuals (HC, N=245) and patients with schizophrenia (SCZ, N=250) or major depressive disorder (MDD, N=240), we extracted power spectrum density and connectivity measures based on 60 second resting-state EEG recordings with 19 channels. The support vector machine models were trained to 1) classify patients with SCZ or MDD and HC individuals, and 2) predict age in HC individuals using ten-by-ten repeated nested-cross validation. The age-predicting model was applied to patient groups to calculate EphysAGE (Electrophysiological Age Gap Estimation) by subtracting chronological age from chronological age. The links between diagnosis, medication, and EphysAGE, i.e., accelerated aging, were then further explored with univariate analyses.

The EphysAGE Model had an explained variance of 46% (MAE=8.7 years, T=14.31, P_1000_<0.001). The patients with SCZ had a significantly higher EphysAGE (mean[SD]=0.61[10.32]) than the patients with MDD (mean[SD]=-1.10[10.49], p=0.04). The classification models discriminated SCZ from HC (Balanced Accuracy, BAC=72.7%, *p*<0.001), MDD from HC (BAC=67.0%, *p*<0.001), and SCZ from MDD individuals (BAC=63.2%, *p*<0.001). Higher EphysAGE was associated with an increased likelihood of being misclassified as SCZ in HC and MDD (ρ_HC_=0.23, *p*<0.001; ρ_MDD_=0.17, *p*=0.01) based on percentile rank scores from the SCZ Model. Moreover, in the Differential Diagnostic Model, higher EphysAGE is positively correlated with being misclassified as SCZ in patients with MDD (ρ_MDD_=0.14, *p*=0.03).

Machine learning models can extract electrophysiological signatures of MDD and SCZ for potential clinical use. However, the impact of aging processes on diagnostic separability calls for timely application of such models, possibly in early recognition settings.

**Disclosure of Interest:**

None Declared

